# Fluorescent Dye Labeling Changes the Biodistribution of Tumor-Targeted Nanoparticles

**DOI:** 10.3390/pharmaceutics12111004

**Published:** 2020-10-22

**Authors:** Patricia Álamo, Victor Pallarès, María Virtudes Céspedes, Aïda Falgàs, Julieta M. Sanchez, Naroa Serna, Laura Sánchez-García, Eric Voltà-Duràn, Gordon A. Morris, Alejandro Sánchez-Chardi, Isolda Casanova, Ramón Mangues, Esther Vazquez, Antonio Villaverde, Ugutz Unzueta

**Affiliations:** 1 Biomedical Research Institute Sant Pau (IIB Sant Pau), Sant Antoni Mª Claret 167, 08025 Barcelona, Spain; PAlamo@santpau.cat (P.Á.); MPallaresL@santpau.cat (V.P.); MCespedes@santpau.cat (M.V.C.); AFalgas@santpau.cat (A.F.); ICasanova@santpau.cat (I.C.); 2 CIBER de Bioingeniería, Biomateriales y Nanomedicina (CIBER-BBN), C/Monforte de Lemos 3–5, 28029 Madrid, Spain; Naroa.Serna@uab.cat (N.S.); laurasanchezgarcia92@gmail.com (L.S.-G.); eric.volta@uab.cat (E.V.-D.); Esther.Vazquez@uab.es (E.V.); 3 Josep Carreras Leukaemia Research Institute (IJC Campus Sant Pau), 08025 Barcelona, Spain; 4 Institut de Biotecnologia i de Biomedicina, Universitat Autònoma de Barcelona, 08193 Bellaterra, Spain; jsanchezqa@gmail.com; 5 Departament de Genètica i de Microbiologia, Universitat Autònoma de Barcelona, 08193 Bellaterra, Spain; 6 ICTA & Cátedra de Química Biológica, Departamento de Química, Instituto de Investigaciones Biológicas y Tecnológicas (IIBYT) (CONICET—Universidad Nacional de Córdoba), FCEFyN, UNC. Av. Velez Sarsfield 1611, X 5016GCA Córdoba, Argentina; 7 Department of Chemical Sciences, School of Applied Science, University of Huddersfield, Queensgate, Huddersfield HD1 3DH, UK; G.Morris@hud.ac.uk; 8 Servei de Microscòpia, Universitat Autònoma de Barcelona, 08193 Bellaterra, Spain; Alejandro.Sanchez.Chardi@uab.cat; 9 Departament de Biologia Evolutiva, Ecologia i Ciències Ambientals, Facultat de Biologia, Universitat de Barcelona, 08028 Barcelona, Spain

**Keywords:** protein nanomaterials, functional materials, self-assembling nanoparticles, fluorescent labeling, biodistribution, targeting

## Abstract

Fluorescent dye labeling is a common strategy to analyze the fate of administered nanoparticles in living organisms. However, to which extent the labeling processes can alter the original nanoparticle biodistribution has been so far neglected. In this work, two widely used fluorescent dye molecules, namely, ATTO488 (ATTO) and Sulfo-Cy5 (S-Cy5), have been covalently attached to a well-characterized CXCR4-targeted self-assembling protein nanoparticle (known as T22-GFP-H6). The biodistribution of labeled T22-GFP-H6-ATTO and T22-GFP-H6-S-Cy5 nanoparticles has been then compared to that of the non-labeled nanoparticle in different CXCR4+ tumor mouse models. We observed that while parental T22-GFP-H6 nanoparticles accumulated mostly and specifically in CXCR4+ tumor cells, labeled T22-GFP-H6-ATTO and T22-GFP-H6-S-Cy5 nanoparticles showed a dramatic change in the biodistribution pattern, accumulating in non-target organs such as liver or kidney while reducing tumor targeting capacity. Therefore, the use of such labeling molecules should be avoided in target and non-target tissue uptake studies during the design and development of targeted nanoscale drug delivery systems, since their effect over the fate of the nanomaterial can lead to considerable miss-interpretations of the actual nanoparticle biodistribution.

## 1. Introduction

Appropriate biodistribution is one of the most sought properties in nanomedicine since selective drug accumulation in target tissues is expected not only to enhance its effectiveness but also to reduce undesired side effects [1,2]. Being this a critical issue, the current trend in the field is to move towards active targeting strategies [3,4,5]. In this sense, one of the key benefits offered by nanoscale structures is the ability to achieve a unique biodistribution pattern by the recruitment of suitable physicochemical properties and the incorporation of effective targeting elements in a single nanoparticle [5]. However, because of their lack of signal emission, it is generically difficult to determine the final fate of developed nanoparticles upon in vivo administration. To address this issue, several nanoparticle-labeling strategies have been developed so far [6,7,8,9].

One of the most used approaches is based on the chemical conjugation of small molecular weight fluorescent probes such as ATTO, Alexa Fluor or Cyanine molecules, in order to monitor their fluorescent signal accumulation in target and non-target tissues upon intravenous administration [10,11,12,13,14,15]. In general, researchers assume that the observed signal distribution pattern is representative of the original nanoparticle behavior. However, whether these labeling processes can significantly alter parental nanoparticle properties and in consequence, their biodistribution, has been so far neglected in most of the studies. In this sense, few studies have already described the effect of encapsulated fluorescent dye leakage over in vitro cell uptake [16], the effect of used conjugation chemistry [17] and the amount of loaded dye molecule [18] over the biodistribution of particles, or the comparison between different fluorescent probes over the tumor accumulation of non-targeted small peptides [19]. Other studies have also suggested a possible influence of fluorescent dye labeling over nanogels [20] or over non-targeted small molecules due to their impact in the final drug size [21]. Recent studies using small molecular weight hybrid radio and fluorescent labeled tracers aimed for image guided surgery have also revealed some differences in the pharmacokinetics and tissue accumulation of tracers depending on fluorescent dye composition and the spacer length [22,23,24,25].

Most of the studies described above analyze the biodistribution of small molecular weight molecules and peptides; however, whether fluorescent dye labeling changes the biodistribution of larger actively targeted nanoscale particles remains still poorly explored, despite its critical value in the design of targeted drug delivery systems.

To address this issue, we have chemically conjugated two different widely known small molecular weight fluorescent probes such as ATTO488 (ATTO) and Sulfo-Cyanine5 (S-Cy5) to the well-characterized T22-GFP-H6 targeted protein nanoparticle. We have then tracked the biodistribution of the original unlabeled T22-GFP-H6 nanoparticle (measuring its intrinsic green fluorescence) and the resulting two labeled variants (T22-GFP-H6-ATTO and T22-GFP-H6-S-Cy5) upon intravenous administration in animal models bearing human, CXCR4+ diffuse large B-cell lymphoma (DLBCL) or colorectal cancer (CRC).

T22-GFP-H6 is a modular fusion protein composed by an N-terminal cationic peptide T22, a potent ligand of the cell surface cytokine receptor CXCR4 [26,27] overexpressed in different human cancer cells [28,29], a fully fluorescent GFP protein scaffold that allows nanoparticle tracking and a C-terminal polyhistidine tag (H6). Both, the C-terminal polyhistidine tag and the N-terminal cationic peptide induce the spontaneous self-assembling of the protein building blocks into regular highly photostable fluorescent nanoparticles of 12 nm [30,31]. T22-GFP-H6 nanoparticles show high architectonic stability in vivo [32] and selectively internalize into CXCR4+ cells in the primary tumor and in macro and micro-metastasis in a disseminated cancer model. Moreover, no significant accumulation in non-target organs upon intravenous injection has been observed in an orthotopic mouse model of colorectal cancer [33,34]. Such a new generation of self-assembling protein-only nanoparticles appears as a paradigmatic example of an active targeting strategy because of its high cell specificity and its outstanding biodistribution selectivity. This has been further confirmed when using this material to transport different biological or chemical drugs, showing a highly selective cytotoxic effect in CXCR4+ target cells [12,35,36,37,38,39]. In fact, T22-GFP-H6 nanoparticles show high clinical relevance as a drug delivery system. In this regard, its covalent binding with the genotoxic antimetabolite Floxuridine (FdU) efficiently and selectively eliminates CXCR4+ cancer stem cells, thus preventing metastases and also inducing the regression of already stablished metastases with no toxicity in non-target tissues [35]. In a similar approach, its covalent binding with Monomethyl auristatin E, a potent tubulin inhibitor, selectively eliminates CXCR4+ leukemic cells showing potent antineoplastic activity in an acute myeloid leukemia animal model [38].

Therefore, labeling such a well-characterized targeted nanoparticle, that display intrinsic GFP fluorescence, with some of the fluorescent probes most widely used in nanomedicine, should allow us to precisely determine the impact of the conjugation process over the original nanoparticle’ biodistribution. Thus, this work could help in making decisions on the suitability of such labeling strategies to study the biodistribution of targeted nanoscale drug delivery systems.

## 2. Materials and Methods 

### 2.1. Protein Production, Purification and Characterization

The protein T22-GFP-H6 was produced as described [40]. In short, a codon-optimized gene encoding T22-GFP-H6 was provided by Geneart (ThermoFisher, Waltham, MA, USA) and the protein was produced in *Escherichia coli* Origami B (Novagen, Merck Millipore, Burlington, MA, USA) at 20 °C overnight upon addition of 0.1 mM Isopropyl-β-d-thiogalactopyranoside (IPTG). Protein nanoparticles were then purified from the soluble fraction of sonicated bacterial cells by IMAC affinity chromatography with HisTrap Chelating HP 5 mL column (GE Healthcare, Chicago, IL, USA) in an ÄKTA pure system (GE Healthcare). Purified proteins were finally dialyzed against sodium carbonate with salt buffer (166 mM NaHCO_3_ 333 mM NaCl, pH = 8). Protein purity was determined by sodium dodecyl sulfate polyacrylamide gel electrophoresis (SDS-PAGE) and Western-blot immunodetection with anti-His monoclonal antibody (Santa Cruz Biotechnology, Dallas, TX, USA). Protein integrity was verified by MALDI-TOF mass spectrometry and final concentration determined by Bradford colorimetric assay in a UV/light spectrophotometer (GE Healthcare) at 595 nm.

Size exclusion chromatography coupled to a Multi Angle Light Scattering (SEC-MALS) was used to calculate the average molar mass of self-assembled T22-GFP-H6 nanoparticles. For that, protein samples were injected in a Superdex 200 increase 10/300 GL column (GE Healthcare) and run in a degassed Nickel enriched sodium carbonate with salt buffer (166 mM NaHCO_3_, 333 mM NaCl, 0.1 mM NiCl_2_, pH = 8). The eluent was monitored by an in-line UV–Vis detector, a Dawn Heleos MALS detector and Optilab rEX RI detector (Wyatt technology Corporation, Santa Barbara, CA, USA). All data were then analyzed with an Astra 6.0.2.9 software (Wyatt technology Corporation), and the average molar masses were calculated in duplicate using both UV (ε: 1.099 mL/(mg.cm)) and RI (dn/dc: 0.185 mL/g) signals as protein concentration sources.

### 2.2. Fluorescent Dye Conjugation

*N*-hydroxysuccinimide ester (NHS-ester) conjugated ATTO488 (Sigma-Aldrich, Saint Louis, MO, USA) and Sulfo-Cyanine 5 (Lumiprobe, Hunt Valley, MD, USA) fluorescent dyes were covalently bound to T22-GFP-H6 protein nanoparticles through exposed external lysines, by ester-amine reaction. For that, an excess of fluorescent probes was incubated in presence of T22-GFP-H6 protein (2:1 dye to protein monomer molar ratio) and sodium carbonate with salt buffer in a one-pot reaction for 1 h and subsequently dialyzed against sodium carbonate with salt buffer in order to remove not reacted fluorescent dye. Conjugation efficiency was then checked by MALDI-TOF mass spectrometry.

### 2.3. Morphometric Characterization

Volume size distribution and Z-potential of T22-GFP-H6 protein nanoparticles and T22-GFP-H6-ATTO nanoconjugates was determined in a Zetasizer Nano ZS (Malvern Instruments, Malvern, UK) by dynamic light scattering (DLS) and electrophoretic light scattering (ELS) respectively at 633 nm. All samples were measured in triplicate. T22-GFP-H6-S-Cy5 nanoconjugates cannot be analyzed by DLS or ELS, since Sulfo-Cyanine 5 fluorescence (excited at 633 nm) prevents reliable light scattering measurements. 

Ultrastructural morphometries of T22-GFP-H6, T22-GFP-H6-ATTO and T22-GFP-H6-S-Cy5 (size and shape) were determined at nearly native state with field emission scanning electron microscopy (FESEM). Drops of 3 µL of diluted samples at 0.3 mg/mL were directly deposited on silicon wafers (Ted Pella Inc., Redding, CA, USA) for 30 s, excess of liquid blotted, air dried and immediately observed without coating with a FESEM Zeiss Merlin (Zeiss, Oberkochen, Germany) operating at 1 kV and equipped with a high resolution in-lens secondary electron detector. In a qualitative approach, representative images of a general fields and nanostructure details were captured at two high magnifications (150,000× and 400,000×). In a quantitative approach, nanoparticles and nanoconjugate average size were analyzed by Image J software (Image J 1.48v, NIH, Bethesda, MA, USA) from FESEM images.

### 2.4. Optical Properties of Nanoparticles and Free Dyes

Excitation and emission spectra of T22-GFP-H6, T22-GFP-H6-ATTO, T22-GFP-H6-S-Cy5 and free ATTO and sulfo-Cy5 dyes were recorded in a Cary Eclipse spectrofluorimeter (Agilent Technologies, Santa Clara, CA, USA). A quartz cell with 10 mm path length and a thermostated holder was used. λem was set at 510 nm (GFP), 523 nm (ATTO) or 662 nm (S-Cy5) for excitation spectra, and λex was set at 450 nm (GFP), 498 nm (ATTO) or 645 nm (S-Cy5) for emission spectra. Dye samples were buffered in carbonate buffer with salt pH 8.

The effect of the free dyes was also studied on the fluorescence properties of T22-GFP-H6. The fluorescence emission spectra of T22-GFP-H6 (0.2 mg/mL) were recorded in the presence of ATTO (0–7 µM) or Sulfo-Cy5 (0–7 µM) (λex = 488 nm). The quenching of T22-GFP-H6 fluorescence induced by Sulfo-Cy5 was evaluated with the Stern–Volmer theory [41]. The linear regression analysis of the Stern–Volmer plot allow us to estimate the Stern–Volmer constant (KSV), according to Equation (1):

(1) $$\frac{Fo}{F}\;=\;1+Ksv\;\times \;\left[Q\right]$$

where *Fo* and *F* correspond to fluorescence intensity in absence or presence of [*Q*] quencher (Sulfo-Cy5).

### 2.5. Selection of Cell Lines for the Generation of In Vitro and In Vivo Models

A human derived Toledo Diffuse Large B-Cell lymphoma (DLBCL) cell line displaying very high CXCR4 overexpression and a scarce stroma was selected as *in vitro* model to carry out the evaluation of receptor specificity of CXCR4-targeted nanoparticles. It was then also used for the generation of DLBCL mouse models. Patient-derived M5 CRC tumor tissue that displays a highly compact solid tumor with a moderate CXCR4 overexpression and a high content of stromal cells that interact with CXCR4+ tumor cells was also used for the generation of CRC mouse models since it maintains the tumor microenvironment observed in humans. 

### 2.6. Cell Culture, Protein Internalization and Competition Assays

Toledo lymphoma cells were incubated in 96-well plates in RPMI medium (Gibco, Rockville, MD, USA) containing 10% of feta bovine serum (Gibco) in humidified atmosphere and 5% CO_2_ at 37 °C for 24 h. Cells were then incubated in presence of 10 nM T22-GFP-H6, T22-GFP-H6-ATTO or T22-GFP-H6-S-Cy5 for 1 h. For competition assays, a potent CXCR4 receptor antagonist AMD3100 (octahydrochloride hydrate, Sigma-Aldrich) was incubated 1 h before sample addition. Incubation plates were then treated with a “harsh” trypsin digestion (1 mg/mL for 15 min) in order to remove externally attached protein, and the internal cell fluorescence was finally analyzed in a FACS Canto flow cytometer (Becton Dickinson, Franklin lakes, NJ, USA) using a 15 mW air-cooled argon ion laser at 488 nm and a D detector (530/30 nm bandpass filter) for GFP and ATTO fluorescence and a laser at 635 nm and a B detector (660/20 bandpass filter) for Sulfo-Cy5 fluorescence. All samples were analyzed in duplicate and free ATTO488 and Sulfo-Cyanine 5 molecules (at 2:1 molar ratio, representing the equivalent excess amount of dye molecule used for the nanoparticle-labeling reaction) were also incubated as controls.

### 2.7. CXCR4 Receptor Binding Affinity

CXCR4 receptor binding affinity in terms of equilibrium dissociation constant (KD) and slope of nonspecific binding (NS) was calculated for CXCR4-targeted T22-GFP-H6, T22-GFP-H6-ATTO and T22-GFP-H6-S-Cy5 nanoparticles in CXCR4+ Toledo Lymphoma cell line by Saturation binding experiments. For that, Toledo cells were incubated in 96-well plates in RPMI medium (Gibco) containing 10% of fetal bovine serum (Gibco) in humidified atmosphere and 5% CO_2_ at 37 °C for 1 h in absence (for total protein binding) or presence (for unspecific protein binding) of the CXCR4 antagonist AMD3100. Cells were then cooled down to 4 °C for 1 h before adding T22-GFP-H6, T22-GFP-H6-ATTO or T22-GFP-H6-S-Cy5 nanoparticles at different concentrations (from 5 nM to 3000 nM) and incubated for an additional hour at 4 °C. Membrane attached protein was finally analyzed in a FACS Canto flow cytometer (Becton Dickinson) using a 15 mW air-cooled argon ion laser at 488 nm and a D detector (530/30 nm bandpass filter) for T22-GFP-H6 and T22-GFP-H6-ATTO and 635 nm laser and B detector (660/20 bandpass filter) for T22-GFP-H6-s-Cy5. All experiments were performed in duplicate and Propidium iodide was added to all samples in order to select living cell population. KD and NS parameters were calculated by binding saturation, one site-total nonlinear regression equation using Graphpad prism 8 software:

$$y\;=\frac{Bmax\;\times \;X}{KD+X}+NS\times \;X+Background$$

where, *y* corresponds to fluorescence signal, *Bmax* is the fluorescence signal at saturation, *X* correspond to sample concentration, *KD* is equilibrium dissociation constant, *NS* is the slope of non-specific binding and Background correspond to system background fluorescence signal. Goodness of fitting for each of the samples is as follows: T22-GFP-H6 (R2: 0.9638), T22-GFP-H6-ATTO (R2: 0.9728) and T22-GFP-H6-S-Cy5 (R2: 0.9889).

### 2.8. Biodistribution in Subcutaneous DLBCL Mouse Model

NOD SCID (NOD.CB17-Prkdcscid/NCrCrl) female mice (4 weeks old) were obtained from Charles River Laboratories. Mice were housed in microisolator units and kept under specific pathogen-free (SPF) conditions with sterile water and food ad libitum. After 1 week of quarantine, NOD SCID mice were subcutaneously injected with Toledo cells in 2 flanks at 10 × 10^6^ cells/100 µL saline solution per flank. Twenty-four days later, when the SC tumors of mice reached approximately 600 mm^3^, 300 µg of T22-GFP-H6 (*n* = 3) nanoparticles and T22-GFP-H6-ATTO (*n* = 3) and T22-GFP-H6-S-Cy5 (*n* = 3) nanoconjugates were injected intravenously (i.v.) to different mice (200 µL). Finally, a mouse was injected i.v. with 200 µL of sodium carbonate with salt buffer as a negative control (*n* = 3). Mice were euthanized after 5 h, and tumors and liver, kidney, lung, pancreas and spleen were excised to measure their fluorescence in IVIS^®^ Spectrum (PerkinElmer, Waltham, MA, USA).

### 2.9. Biodistribution in Subcutaneous Colorectal Cancer Mouse Model

Five-week-old female Swiss Nu/Nu mice, weighing 18–20 g (Charles River, Wilmington, MA, USA) were used and maintained in specific pathogen-free conditions. To generate the subcutaneous (SC) mouse model, we implanted subcutaneously 10 mg of the patient-derived M5 colorectal (CRC) tumor tissue from donor animals in the mouse subcutis. When tumors reached approximately 500 mm^3^, mice received one single 300 μg i.v. bolus of T22-GFP-H6 (*n* = 3), T22-GFP-H6-ATTO (*n* = 3) or T22-GFP-H6-S-Cy5 (*n* = 2) in sodium carbonate with salt buffer. Control animals received the same buffer (*n* = 2), 0.25 μg of free ATTO488 (*n* = 2) or 0.0347 μg of free Sulfo-Cy5 (*n* = 2). At 5 h, mice were euthanized and subcutaneous tumors and organs (liver, kidney, lung, pancreas and spleen) were collected. Biodistribution of T22-GFP-H6 nanoparticles, and ATTO488- and Sulfo-Cy5-labeled nanoconjugates in tumor and non-tumor organs was determined by measuring the emitted fluorescence in ex vivo tissue sections (3 mm thick), using the IVIS^®^ Spectrum (Perkin Elmer) platform.

All in vivo experiments and procedures in both colorectal cancer and DLBCL mouse models were approved and conducted in accordance with guidelines by the institutional animal Ethics Committee of Hospital de Sant Pau (Barcelona, Spain). Furthermore, based on the results obtained in previous biodistribution assays, we used the sample size and power calculator (GRANMO v. 7.12, Institut Municipal d’Investigació Mèdica IMIM, Barcelona, Spain) with the two-sided test for two independent means to determine sample size. The results established that the minimum number of animals per group to be used in both models was 2. Time points for the biodistribution studies were selected based on previous assays. In them, T22-GFP-H6 showed the peak of protein uptake in tumor at 5 h post-injection, and fluorescence was then progressively reduced by proteolysis leading to complete degradation of the nanoparticle [42]. To select the organs to be included in the study, a preliminary ex vivo screening of all organs was performed by IVIS^®^ Spectrum imaging platform. Those showing a fluorescence emission over the tissue background auto-fluorescence were selected.

### 2.10. Ex Vivo Biodistribution Fluorescent Image Analysis

The fluorescent signals (FLI) measured using the IVIS^®^ Spectrum (Perkin Elmer) correlate with the amount of administered protein accumulated in each tissue. Emitted FLI signals were first digitalized, displayed as a pseudocolor overlay and normalized by the organ size to be expressed as radiant efficiency ((p/sec/cm^2^/sr)/μW/cm^2^). FLI values were calculated subtracting the auto-fluorescence from the negative control and finally expressed as tissue/tumor ratio to compare the biodistribution between different nanoparticles. Data were also expressed as a percentage of distribution among the analyzed organs. The excitation–emission filters used to measure the FLI of each nanoparticle and nanoconjugate were selected in a preliminary experiment where different sets of bandpass (BP) filters were tested and the spectral regions where samples showed the maximum fluorescence signal selected: T22-GFP-H6 was excited by 465/30 BP filter (450–480 nm) and recorded with 520/20 BP filter (510–530 nm). T22-GFP-H6-ATTO was excited by 500/30 BP filter (485–515 nm) and recorded by 540/20 BP filter (530–550 nm). Finally, T22-GFP-H6-S-Cy5 was excited by 640/30 BP filter (625–655 nm) and monitored by 700/20 BP filter (690–710 nm).

### 2.11. Statistical Analysis

Pairwise comparisons between sample groups in *in vitro* experiments were determined by Student t-tests using SigmaPlot 10.0 (Systat software Inc., Berkshire, UK). For the in vivo experiments *t*-test and Mann–Whitney U-tests were used to analyze differences in biodistribution between nanoparticle and nanoconjugate groups for each tested organ. Differences between groups were considered significant at *p* < 0.05. All in vivo statistical analyses were performed using SPSS version 22.0 package (IBM, Armonk, NY, USA), and values were expressed as mean ± standard error of the mean ($\bar{X}$ ± SEM). 

## 3. Results

**Figure 1:**
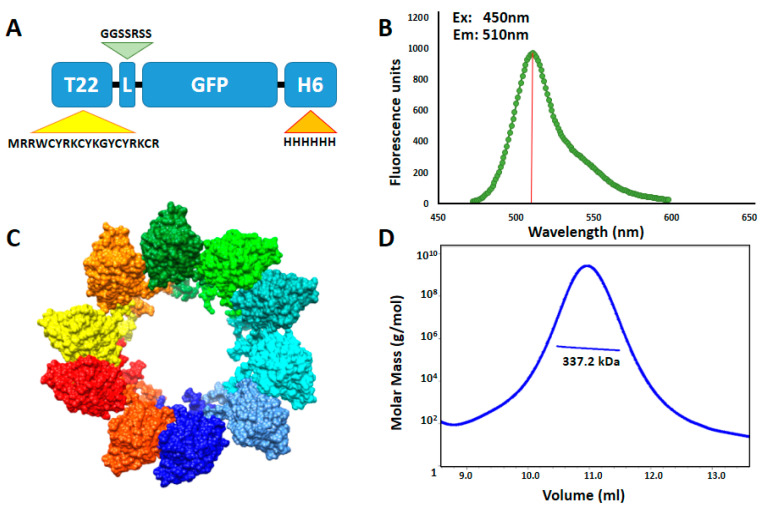
T22-GFP-H6 nanoparticle characterization. (**A**) Modular organization of T22-GFP-H6 protein building blocks. T22 is a CXCR4 specific cationic tag derivative of poliphemusin II protein from horseshoe crab [26]. L is a linker commonly used in phage display and H6 corresponds to polyhistidine architectonic tag. Relative length of the modules is only indicative. (**B**) Protein intrinsic fluorescence emission spectrum upon excitation at 450 nm wavelength. Red bar indicates fluorescence emission maximum at 510 nm. (**C**) In silico supramolecular organization model of protein building blocks into toroid-like nanoparticles. Each monomer is represented in a different color. Modified from [43] with permission of John Wiley and Sons. (**D**) Average molar mass distribution of T22-GFP-H6 nanoparticles determined by size exclusion chromatography coupled to multi angle light scattering (SEC-MALS).

**Figure 2:**
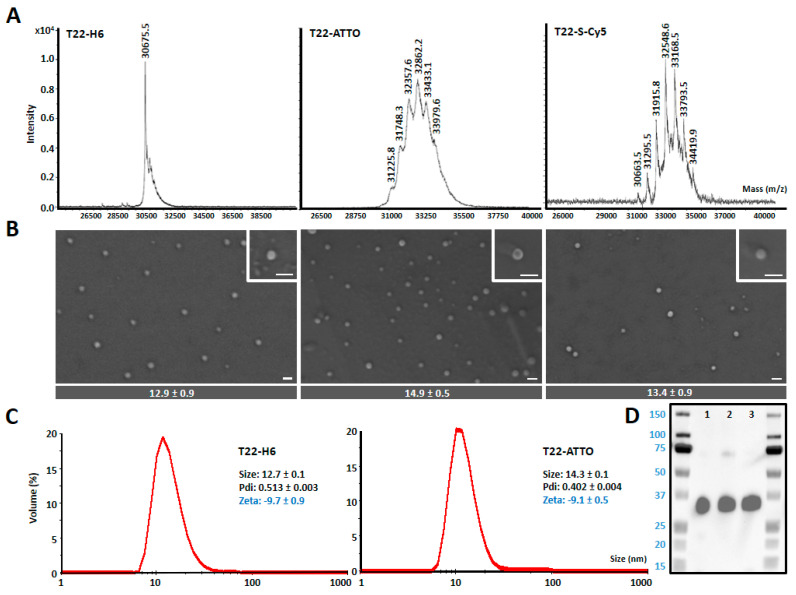
Nanoarchitecture of nanoconstructs. (**A**) MALDI-TOF mass spectrometry of T22-GFP-H6 (T22) nanoparticles and T22-GFP-H6-ATTO (T22-ATTO) and T22-GFP-H6-S-Cy5 (T22-S-Cy5) nanoconjugates. Each peak over 30.6 kDa (T22-GFP-H6) corresponds to the addition of a single ATTO488 or Sulfo-Cy5 molecule, respectively. (**B**) Representative electron microscopy (FESEM) images of the three nanoconstructs (nanoparticles and nanoconjugates) presented in two magnifications (see insets), equivalent in all images. Scale bars indicate 30 nm. At the bottom, quantitative average size of nanoconstructs is determined by image analysis and shown as mean ± standard error. (**C**) Volume size distribution (Size) and Z-potential (Zeta) of protein nanoparticles measured by light scattering. T22-GFP-H6-S-Cy5 sample cannot be analyzed since S-Cy5 fluorescence interferes with light scattering measurements. Pdi indicates polydispersion index. (**D**) Western blot analysis of T22-GFP-H6 (1), T22-GFP-H6-S-Cy5 (2) and T22-GFP-H6-ATTO (3) immunodetected by an anti-GFP monoclonal antibody.

T22-GFP-H6 is a fully fluorescent 30.6 kDa modular protein that spontaneously self-assembles into 12 nm toroid-like structure containing around 10 copies of the polypeptide [43] (Figure 1). Additional nanoparticle labeling with ATTO488 and Sulfo-Cy5 fluorescent probes was achieved by chemical conjugation through the free lysine-amine groups of the protein. These fluorescent molecules were selected since being widely used labeling molecules, they show different biophysical properties (Supplementary Figure S1) [44]. This process resulted in fully stable protein nanoconjugates that efficiently incorporated fluorescent molecules having the expected Poisson dye load distribution in this type of conjugation [45] (Figure 2A) and a fluorescence emission corresponding to the conjugated dye molecules (Supplementary Figure S2). In general terms, nanoparticle labeling did not affect protein integrity (Figure 2D), surface charge or original nanostructure as assessed by DLS, ELS (Figure 2C) and FESEM (Figure 2B). However, an intrinsic GFP fluorescence reduction was observed in T22-GFP-H6-S-Cy5 nanoconjugates, due to the quenching induced by S-Cy5 moieties (Supplementary Figure S3). In consequence, further *in vitro* and in vivo nanoparticles analysis was followed by green fluorescence emission for T22-GFP-H6 (GFP fluorescence) and T22-GFP-H6-ATTO (GFP and ATTO fluorescence) and by red fluorescence emission for T22-GFP-H6-S-Cy5 (S-Cy5 fluorescence). The size of T22-GFP-H6-S-Cy5 could only be determined by image analysis of high-resolution electron microscopy since Cy5 fluorescence also interfered with DLS and ELS measurements.

**Figure 3:**
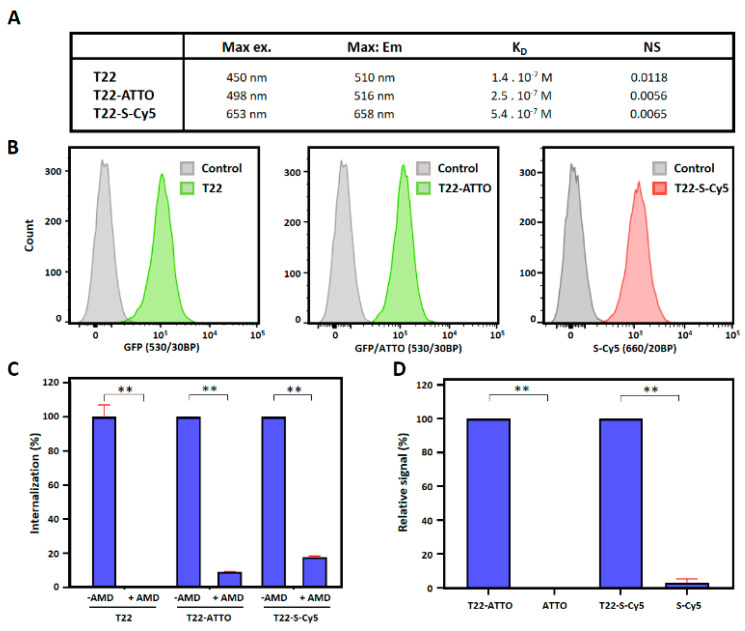
CXCR4-mediated cell internalization of nanoconstructs. (**A**) Biophysical properties of T22-GFP-H6 (T22), T22-GFP-H6-ATTO (T22-ATTO) and T22-GFP-H6-S-Cy5 (T22-S-Cy5) nanoparticles. (**B**) T22-GFP-H6 (T22) nanoparticles, and labeled T22-GFP-H6-ATTO (T22-ATTO) and T22-GFP-H6-S-Cy5 (T22-S-Cy5) nanoparticles internalization in cultured CXCR4+ Toledo cells monitored by flow cytometry upon 1 h of exposure. Grey peak overlays show control cells auto-fluorescence. 530/30BP indicates 515–545 nm bandpass filter, and 660/20BP indicates 650–670 nm bandpass filter. (**C**) CXCR4-mediated internalization in presence and absence of the CXCR4 specific antagonist AMD3100, upon 1 h of exposure. (**D**) Background fluorescence signal upon incubation of free ATTO488 (ATTO) and Sulfo-Cy5 (S-Cy5) in Toledo cells for 1 h. Fluorescence signal of free samples is shown relative to their respective nanoconjugate signals. Significant differences at *p* < 0.01 are indicated as **.

We then tested whether fluorescent labeling could significantly disturb the CXCR4-mediated endosomal cell internalization of nanoparticles in CXCR4+ human Toledo lymphoma cells. In this context, all labeled and non-labeled nanoparticles showed very efficient cell internalization upon in vitro incubation (Figure 3B) although the receptor equilibrium dissociation constant (KD) was higher in labeled than in unlabeled nanoparticles (Figure 3A, Supplementary Figure S4). In addition, internalization could be, in all cases, efficiently blocked when it was competed with the CXCR4 specific antagonist AMD3100 [46] showing high receptor specificity in all samples (Figure 3C). Excess amounts of free ATTO488 and sulfo-Cy5 were also incubated in the presence of Toledo cells in order to test unspecific membrane affinity of free fluorescent molecules. Results showed only background fluorescence signal for both fluorochromes revealing no significant interaction with cells (Figure 3D).

Once proved that nanoparticle labeling did not alter their receptor specificity for internalization, we studied the proteolytic and structural stability of nanoparticles in serum. In this sense, no protein degradation was detected upon incubation of labeled and unlabeled nanoparticles in human serum for 24 h. No significant alterations in nanoparticles hydrodynamic size were either detected, suggesting strong structural stability and no interactions with serum proteins (Supplementary Figure S5).

**Figure 4:**
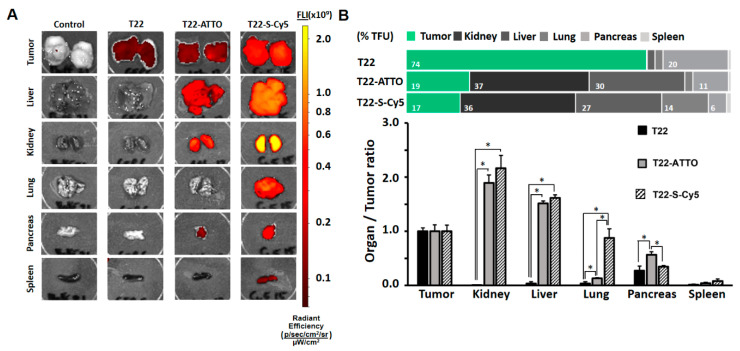
Biodistribution in subcutaneous DLBCL mouse model. (**A**) Images of tumor and non-target organ sections of DLBCL mouse model at 5 h upon administration of buffer (Control), T22-GFP-H6 (T22) nanoparticles or labeled T22-GFP-H6-ATTO (T22-ATTO) and T22-GFP-H6-S-Cy5 (T22-S-Cy5) nanoconjugates monitored by IVIS spectrum and displayed as pseudocolor overlay expressing radiant efficiency. T22-GFP-H6 was excited at 450–480 nm and recorded at 510–530 nm bandpass; T22-GFP-H6-ATTO was excited at 485–515 nm and monitored at 530–550 nm bandpass, and T22-GFP-H6-S-Cy5 was excited at 625–655 nm and monitored at 690–710 nm bandpass. (**B**) Relative tissue accumulation of nanoparticles at 5 h post-administration expressed as organ to tumor ratio. Upper horizontal bars represent percentages of total fluorescence uptake (% TFU) in different organs. All data are presented as mean ± standard error. Significant differences at *p* < 0.05 are indicated as *.

We then finally moved forward to in vivo biodistribution studies in a Toledo cell line-derived subcutaneous CXCR4+ DLBCL mouse model to determine if the labeling of nanoparticles could significantly modify their original biodistribution pattern. The intravenous administration of control T22-GFP-H6 nanoparticles, which shows enough intrinsic fluorescent for ex vivo tracking, resulted in their stable accumulation in target tumor cells at 5 h post-administration (74% ± 5% TFU). In contrast, they showed very low uptake in main non-target organs such as spleen, liver, kidney or lung (range 0.2–3% TFU), with only moderate signal in pancreas (20% ± 6% TFU) (Figure 4, Supplementary Table S1) as previously reported in similar biodistribution studies [34]. In contrast, the administration of labeled T22-GFP-H6-ATTO or T22-GFP-H6-S-Cy5 nanoconjugates resulted in a dramatically different distribution pattern, showing a strong reduction in tumor uptake being in a similar range in both cases (17% ± 2%–19% ± 2% TFU) and an unexpected accumulation in non-target organs, being especially significant in liver (range: 26.6% ± 0.9%–29.5% ± 0.9% TFU) and kidney (range: 36% ± 4%–37% ± 3% TFU) (Figure 4, Supplementary Table S1). 

**Figure 5:**
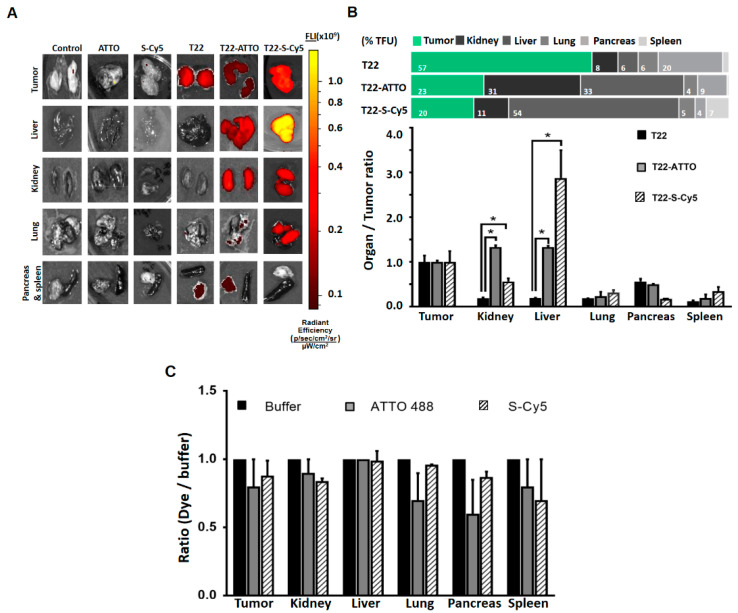
Biodistribution in colorectal cancer model. (**A**) Images of tumor and non-target organ sections of colorectal cancer mouse model at 5 h upon administration of buffer (Control), free ATTO488 (ATTO) and Sulfo-Cy5 (S-Cy5), T22-GFP-H6 (T22) nanoparticles or labeled T22-GFP-H6-ATTO (T22-ATTO) and T22-GFP-H6-S-Cy5 (T22-S-Cy5) nanoconjugates, monitored by the IVIS spectrum and displayed as pseudocolor overlay expressing radiant efficiency. T22-GFP-H6 was excited at 450–480 nm and recorded at 510–530 nm bandpass; T22-GFP-H6-ATTO was excited at 485–515 nm and monitored at 530–550 nm bandpass, and T22-GFP-H6-S-Cy5 was excited at 625–655 nm and monitored at 690–710 nm bandpass. (**B**) Relative tissue accumulation of nanoparticles expressed as organ to tumor ratio. Upper horizontal bars represent the percentages of total fluorescence uptake (% TFU) in different organs (**C**) Free ATTO488 (ATTO) and Sulfo-Cy5 (S-Cy5) tissue accumulation upon 5 h of administration expressed as fluorochrome to buffer ratio. All data are presented as mean ± standard error. Significant differences at *p* < 0.05 are indicated as *.

The same comparative biodistribution study was performed in an alternative M5 tumor cell line-derived subcutaneous CXCR4+ colorectal cancer mouse model in order to determine if the previously observed biodistribution changes were replicated in a completely different tumor model. Similarly, as found in the DLBCL model, the administration of the original unlabeled T22-GFP-H6 nanoparticles resulted in a high accumulation in tumor (57% ± 7% TFU) with only a moderate accumulation in pancreas at 5 h post-administration (20% ± 3% TFU). However, the administration of T22-GFP-H6-ATTO and T22-GFP-H6-S-Cy5 also resulted in a drastic reduction of tumor uptake to the range of 20% ± 8%–22.9% ± 0.8% TFU, and in a significant accumulation of labeled nanoparticles in liver (range: 32.5% ± 0.9%–54% ± 12% TFU) and kidney (range: 11% ± 0.5%–30% ± 1% TFU) (Figure 5A,B, Supplementary Table S2).

To discard possible unspecific signal accumulation produced by free fluorescent dye molecules coming from nanoconjugate leakage in blood, we intravenously administered an equivalent fluorescent amount of free ATTO488 or Sulfo-Cy5 molecules that resulted in no accumulation in tumor or any other organ (Figure 5C).

## 4. Discussion

Developing tracking strategies for nanoparticles is a key issue in nanomedicine and especially in targeted drug delivery systems. However, whether the processes for labeling drugs or their vehicles can significantly alter their tissue accumulation pattern needs to be considered since disrupted distributions can lead to misinterpretations of the material original behavior. In this sense, we have shown here how some of the most widely used labeling molecules such as small molecular weight fluorescent probes can significantly change the biodistribution of a very well characterized active targeted protein nanoparticle namely T22-GFP-H6 [33,34]. We chose CXCR4+ tumor models since they show high receptor overexpression allowing highly selective nanoparticle targeting. In this sense, GFP is an intrinsically fluorescent and biologically neutral protein that does not interact or accumulate in body tissues upon i.v administration, showing only the expected fast renal clearance due to its small molecular size (<6 nm) [32]. However, when self-assembled as 12 nm nanoparticles (above the renal filtration cut-off) and actively targeted to CXCR4+ cells with T22, it shows absence of renal clearance and outstanding tumor specificity with most of the administered material accumulated in target tissue in different models of solid tumors and hematological malignances [32,34,42]. The chemical conjugation of ATTO488 or Sulfo-Cy5 dramatically reduced the tumor targeting capacity of T22-GFP-H6 nanoparticles showing increased accumulation of the nanomaterial in non-target organs such as liver or kidney, in two different CXCR4+ tumor mouse models (Figure 4 and Figure 5) that differ in their CXCR4 expression level (Supplementary Figure S6) and mouse strain. More specifically, the relative CXCR4+ tumor accumulation significantly decreased from around 60–75% to 15–20% in labeled nanoparticles while their off-target tissue uptake dramatically increased in both models. This unspecific tissue accumulation was particularly significant in liver and kidney (Figure 4B and Figure 5B). Liver/tumor accumulation ratio significantly increased from below 0.2 in T22-GFP-H6 to around 1.5 in labeled nanoparticles. This increase was particularly high for T22-GFP-H6-S-Cy5 in the CRC model. Similar behavior was observed in kidney where relative kidney/tumor uptake ratio also increased from below 0.2 to around 1.5–2.0 in both models, being more moderate again in the case of T22-GFP-H6-S-Cy5 in the CRC model (Figure 4B and Figure 5B). The mild differences observed between the two tumor models could be related to their differences in CXCR4 overexpression level (higher in the Toledo DLBCL model, Supplementary Figure S6), but also, to the use of different mouse strains to develop them, since Swiss nude mice (used for the M5 CRC model) are more immunocompetent than NOD SCID mice (required for developing Toledo DLBCL model). Thus, the presence or absence of immune cells could also contribute to modulate nanoparticles interaction and retention in the studied organs.

Our results are in tune with other reported biodistribution patterns where different types of nanoparticles, including carbon nanotubes [47], self-assembling bacterial nanotoxins [12] or chitosan, lipidoid and latex nanoparticles [14,15,48], were similarly labeled with small molecular weight fluorescent dyes such as Rhodamines, Cyanines or ATTO molecules for in vivo tracking purposes. In all these experiments, strong liver or kidney accumulation was observed upon in vivo administration. However, none of those studies considered this biodistribution pattern a direct result of particle labeling. Other experiments using different molecular weight proteins with natural affinity for liver accumulation, such as BSA or IgG-s, have also reported increased liver uptake upon different fluorescent dye molecules conjugation [21]. This is a particularly critical issue since hepatic uptake and toxicity of administered drugs represent severe concerns in cancer therapies. This undesired hepatic damage occurs even in actively targeted nanoconjugates including antibody drug conjugates (ADC) [49,50,51,52], for which liver uptake has shown to be especially enhanced when transporting drugs at high drug antibody ratios (DAR) [53]. On the other hand, artifact particle accumulation could induce misleading interpretations about the biodistribution of the drug, which is critical in the design of targeted drug delivery systems.

Observed changes in nanoparticle biodistribution patterns could not be related with their supramolecular structure properties since no changes over nanoparticle size, shape or surface charge was observed upon conjugation (Figure 2B,C). Nanoparticle’s specificity for the CXCR4 receptor was neither negatively affected as seen in the competition assay where highly CXCR4-receptor dependent internalization was reported for all samples (Figure 3C). In addition, saturation binding experiments also showed lower non-specific binding (NS) for labeled nanoparticles than for parental T22-GFP-H6 (Figure 3A). The receptor binding affinity, however, was lower in labeled nanoparticles than in parental non-labeled ones (KD T22-GFP-H6 < KD labeled T22-GFP-H6), although in the same order of magnitude (Figure 3A), which could in some extend contribute to observed behavior. In this sense, studies based in radiolabeled small molecules, single chain Fv antibodies or monoclonal antibodies using variants with binding affinities at different orders of magnitude, have reported some differences in tumor uptake in vivo [54,55,56]. However, biodistribution being the result of a multifactorial process, it is not clear how dissociation constant (KD) can influence the tumor uptake of high molecular weight multivalent targeted nanoparticles, since higher affinities have shown to favor the tumor uptake of small molecules plateauing at certain level [54] while restricting the localization and tumor penetration of antibodies [55,56]. In this regard, it has been described that combinatorial binding entropy, related with the equilibrium between the attractive and repulsive contribution of multiple ligands and receptors, determines the binding in multivalent constructs, resulting in much sharper response to receptor density than monovalent systems, which is called super selectivity [57,58,59]. In this sense, we observe higher selectivity and tumor targeting capacity of parental T22-GFP-H6 nanoparticles in DLBCL mouse model, which overexpress higher CXCR4 receptor level (Supplementary Figure S6), than in CRC mouse model. Therefore, the observed differences in KD values could be a consequence of a displacement in the equilibrium between those attractive and repulsive forces in the multivalent T22-CXCR4 interaction.

In this regard, the conjugation chemistry used for T22-GFP-H6 labeling does not seem to be the problem, since the covalent binding of small molecular weight drugs such as FdU, using the same lysine/amine chemistry, resulted in a perfectly functional T22-GFP-H6-FdU nanoconjugate with highly selective tumor accumulation [35].

On the other hand, the contribution of fluorescent dye leakage from labeled T22-GFP-H6-ATTO and T22-GFP-H6-S-Cy5 nanoconjugates and their subsequent unspecific tissue accumulation in the observed biodistribution pattern are completely discarded since the lysine-amine covalent binding of fluorochromes to the protein, which is widely used in nanomedicine including marketed products such as ADC Kadcyla [60], results in a very stable conjugation with low leaking rate. In this respect, no significant fluorochrome leakage was detected in labeled nanoconjugates, being in both cases less than 10% (1.1% for S-Cy5 and 7.1% for ATTO) of conjugated material released from the nanoparticle upon 24 h of exposure to human serum (Supplementary Figure S7). Indeed, the administration of free ATTO488 or Sulfo-Cy5 molecules, whose molecular weights are below the renal excretion cut-off (0.98 kDa and 0.78 kDa, respectively), resulted in fast renal clearance and undetectable accumulation either in tumor or in any other organ 5 h after their direct injection in mice (Figure 5C). Therefore, the low amount of dissociated fluorophore molecules are expected to be rapidly cleared in the kidney and excreted in the urine without recirculation in blood, while the accumulation in the liver or the kidney is in form of nanoconjugate, which because of its high molecular weight (337 kDa, Figure 1) and size (12.7 nm, Figure 2) recirculate in blood for long period of time.

The physicochemical properties of conjugated fluorescent molecules could also be an additional factor driving the fate of nanoparticles. In fact, alternative distribution patterns have been already reported for untargeted peptides when attached to several fluorescent probes with different physicochemical properties [19]. In this context, ATTO488 is a water-soluble small molecular weight fluorescent dye with an excitation maximum at 500 nm and with an emission peak at 520 nm that shows no affinity for unspecific membrane interactions. On the other hand, Sulfo-Cy5 is a slightly more hydrophobic fluorescent dye with excitation maximum at 646 nm and emission peak at 662 nm that shows moderate membrane interaction activity [44]. Both fluorescent molecules perfectly kept the described emission spectra in aqueous sodium carbonate solution although a slight fluorescence shift (−4 nm) was observed in the conjugated versions (Supplementary Figures S1 and S2). Interestingly, the hydrophobicity of fluorescent molecules has been related with unspecific interactions and liver accumulation [19,61].

In this sense, although the reported hydrophobicity (log D value) and the membrane interaction factor (MIF value) are significantly different for both ATTO and Sulfo-Cy5 molecules [44] (Supplementary Figure S1), none of the free molecules showed significant interaction with Toledo cells (Figure 3D) in vitro nor unspecific accumulation in any tissue in vivo (Figure 5C). Regarding labeled nanoparticles, both T22-GFP-H6-ATTO and T22-GFP-H6-S-Cy5, present similar receptor specificity in cell culture (Figure 3C) and similar loss of targeting capacity in both human CXCR4+ cancer mouse models in vivo (Figure 4 and Figure 5). Only moderate differences in liver/kidney ratio have been observed between both labeled nanoparticles in the CRC model (Figure 5). Therefore, the hydrophobicity of fluorescent molecules itself does not correlate with the general biodistribution changes observed in vivo, although it could contribute to modulate it.

Overall, as the exact mechanisms involved in the observed modification of biodistribution are still unclear, alternative tracking strategies such as nuclear imaging or labeling molecule encapsulation need to be considered, while new fluorescent dye molecules aimed at having low or null impact on nanoparticle biodistribution and targeting are being developed. In this sense, the incorporation of radioisotopes in the protein building block is still considered the best alternative to study their biodistribution since it maintains nanoparticles molecular structure and physicochemical properties while allowing its radioactive emission based tracking as already seen in ADCs [62,63,64]. Meanwhile, the implementation of site-directed conjugation strategies, already in development for ADCs [65], will allow to further investigate the effect of the amount and the dye load localization over actively targeted nanoscale particles in the near future.

## 5. Conclusions

Tracking-oriented labeling of nanoparticles can significantly alter their in vivo pharmacokinetic profiles, which can induce misleading interpretation of target specificity, as demonstrated here by the use of small molecular weight fluorescent molecules. The chemical conjugation of widely used fluorescent probes to a very well characterized actively targeted nanoparticle with highly specific biodistribution profile, reduces its tumor targeting capacity while dramatically increasing its liver and kidney accumulation. Although the exact mechanisms involved in this multifactorial process are still unclear, this is a highly relevant issue that needs to be considered in the design of nanoparticles as targeted drug delivery systems. In this sense, different labeling strategies need to be evaluated in order to ensure that particle pharmacokinetic properties are not modified during the process inducing dramatic misinterpretation of their tissue biodistribution.

## Supplementary Materials

The following are available online at https://www.mdpi.com/1999-4923/12/11/1004/s1, Figure S1. Biophysical properties of free fluorescent dyes, Figure S2. Comparative analysis of fluorescence emission spectra, Figure S3. Fluorescence dye mediated GFP quenching analysis, Figure S4. Nanoparticles specific binding to the CXCR4 receptor, Figure S5. Nanoparticles stability in human serum, Figure S6. CXCR4 expression in DLBCL and CRC models, Figure S7. Fluorescent dye leakage in human serum, Table S1. Biodistribution in subcutaneous DLBCL mouse model 5 h post administration represented as percentages of total fluorescence uptake (% TFU) in different organs, Table S2. Biodistribution in subcutaneous CRC mouse model 5 h post administration represented as percentages of total fluorescence uptake (% TFU) in different organs.
