# Supplementary material for: Fluorescent Dye Labeling Changes the Biodistribution of Tumor-Targeted Nanoparticles
Source: Pharmaceutics. 2020 Oct 22;12(11):1004. doi: 10.3390/pharmaceutics12111004 (PMC7690626; doi:10.3390/pharmaceutics12111004)
Supplement: Supplementary file 1 [file pharmaceutics-12-01004-s001.pdf]

pharmaceutics

Supplementary Materials: Fluorescent Dye Labeling Changes the Biodistribution of Tumor-targeted Nanoparticles

Patricia Álamo, Victor Pallarès, María Virtudes Céspedes, Aïda Falgàs, Julieta M. Sanchez, Naroa Serna, Laura Sánchez-García, Eric Voltà-Duràn, Gordon A. Morris, Alejandro Sánchez-Chardi, Isolda Casanova, Ramón Mangues, Esther Vazquez, Antonio Villaverde and Ugutz Unzueta

Nanoparticles stability in front of human serum:

T22-GFP-H6, T22-GFP-H6-ATTO and T22-GFP-H6-S-Cy5 nanoparticles were incubated in front of human serum (Sigma) for different times (0, 2, 5 and 24 h) at 37 °C and a final concentration of 1mg/ml. Then, their nanostructure stability was analyzed by Dynamic Light Scattering and their proteolytic stability was also determined by western-blot immunostaining using a monoclonal anti-His antibody (Santa Cruz Biotechnology/sc-57598/batch: B1916)). T22-GFP-H6-S-Cy5 nanoparticles could not be analyzed by DLS since Sulfo-Cyanine 5 fluorescence (exited at 633 nm) prevent reliable light scattering measurements.

Fluorimeter and Fluorescent dye conjugation stability in front of human serum:

Fluorescence emission spectra of T22-GFP-H6, T22-GFP-H6-ATTO, T22-GFP-H6-S-Cy5 and free ATTO488 and sulfo-Cy5 molecules were measured in a Varian Cary Eclipse fluorescence spectrophotometer (Agilent Technologies). GFP and ATTO488 molecules were excited at 488nm and their emission recorded at 500–600 nm range. S-Cy5 molecules were exited at 630nm and their emission recorded at 650–700 nm range.

Stability of ATTO488 and sulfo-Cy5 conjugation in presence of human serum was assessed through fluorescence spectrometry. For that, both T22-GFP-H6-ATTO and T22-GFP-H6-S-Cy5 were incubated at a final concentration of 0.5mg/ml in presence of human serum (S2257, Sigma) for 24 h at 37 °C. Then, samples were dialyzed against their storage carbonate with salt buffer (166 mM $NaHCO_3$ + 333 mM NaCl, pH 8) for 2 h at room temperature to remove the free dye molecules that might be released from the nanoparticle. Additionally, same amount of both free ATTO488 and Sulfo-Cy5 were also dialyzed as a positive control (considering the conjugation ratio of 1:2). Finally, dialysis buffers fluorescence was measured in a Varian Cary Eclipse fluorescence spectrophotometer (Agilent Technologies) to test the presence of fluorescent dye molecules coming from the leakage of T22-GFP-H6-ATTO and T22-GFP-H6-S-Cy5 nanoconjugates or controls. ATTO488 molecules were excited at 488 nm and their emission recorded at 523 nm and Sulfo-Cy5 molecules were excited at 630 nm and their emission recorded at 663 nm.

Immunohistochemistry (IHC) analysis of CXCR4 expression:

To determine CXCR4 expression in tumors models, samples derived from M5 and Toledo subcutaneous tumors were fixed, and paraffin-embedded to determine the levels of tumor cells expressing CXCR4 receptor using IHC with an anti-CXCR4 antibody (1:200, pH high. Abcam, UK) as previously described (Céspedes *et al*, 2014). CXCR4 expression was evaluated using Cell∧D Olympus software (v3.3.).

A

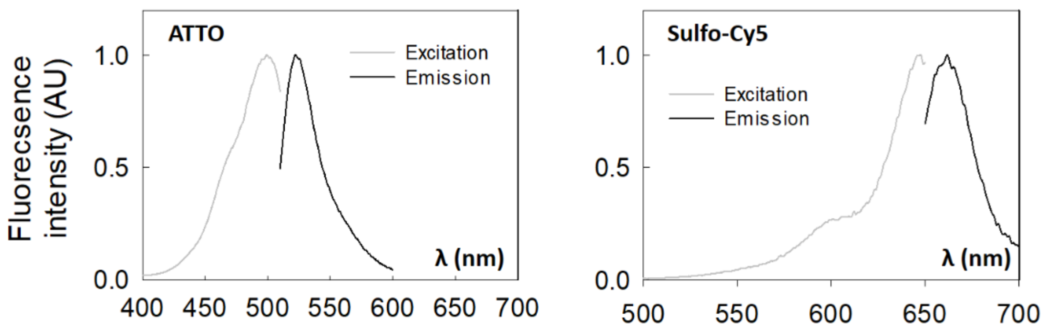

B

	Max ex.	Max: Em	LogD	MIF
**ATTO488**	498 nm	520 nm	-4.67	0.0007
**Sulfo-Cy5**	645 nm	662 nm	1.65	0.31

Figure S1. Biophysical properties of free fluorescent dyes. **A**) Excitation and emission spectra of free ATTO and Sulfo-Cy5 fluorescent dyes. **B**) Summary of biophysical properties of free ATTO and Sulfo-Cy5 dyes. Log D and MIF data are extracted from [1].

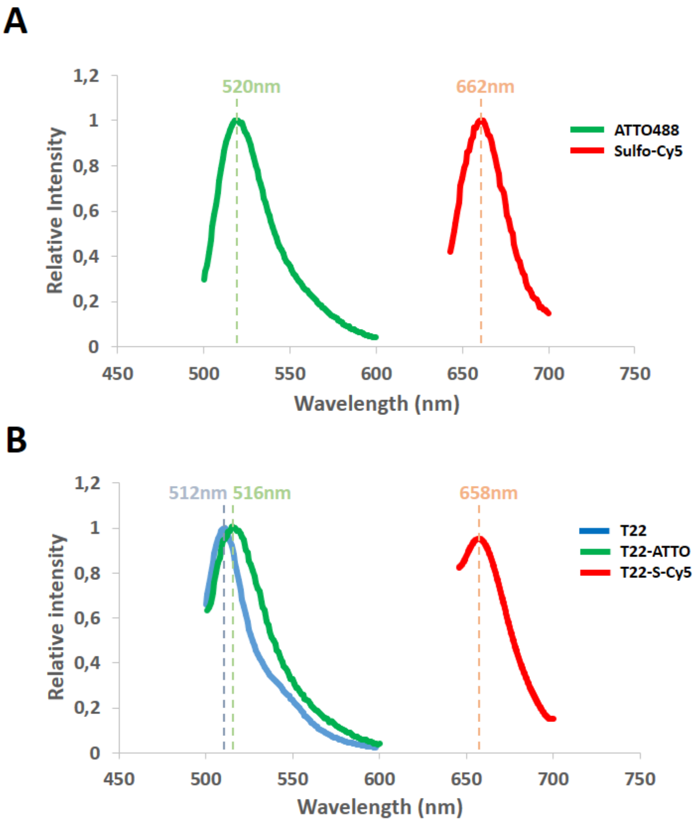

Figure S2. Comparative analysis of fluorescence emission spectra: **A**) Fluorescence emission spectra of free ATTO488 and Sulfo-Cy5 molecules. **B**) Fluorescence emission spectra of non-labeled T22-GFP-H6 (T22) and labeled T22-GFP-H6-ATTO (T22-ATTO) and T22-GFP-H6-S-Cy5 (T22-S-Cy5) nanoparticles. Dashed lines indicate fluorescence emission maximum wavelength for each sample.

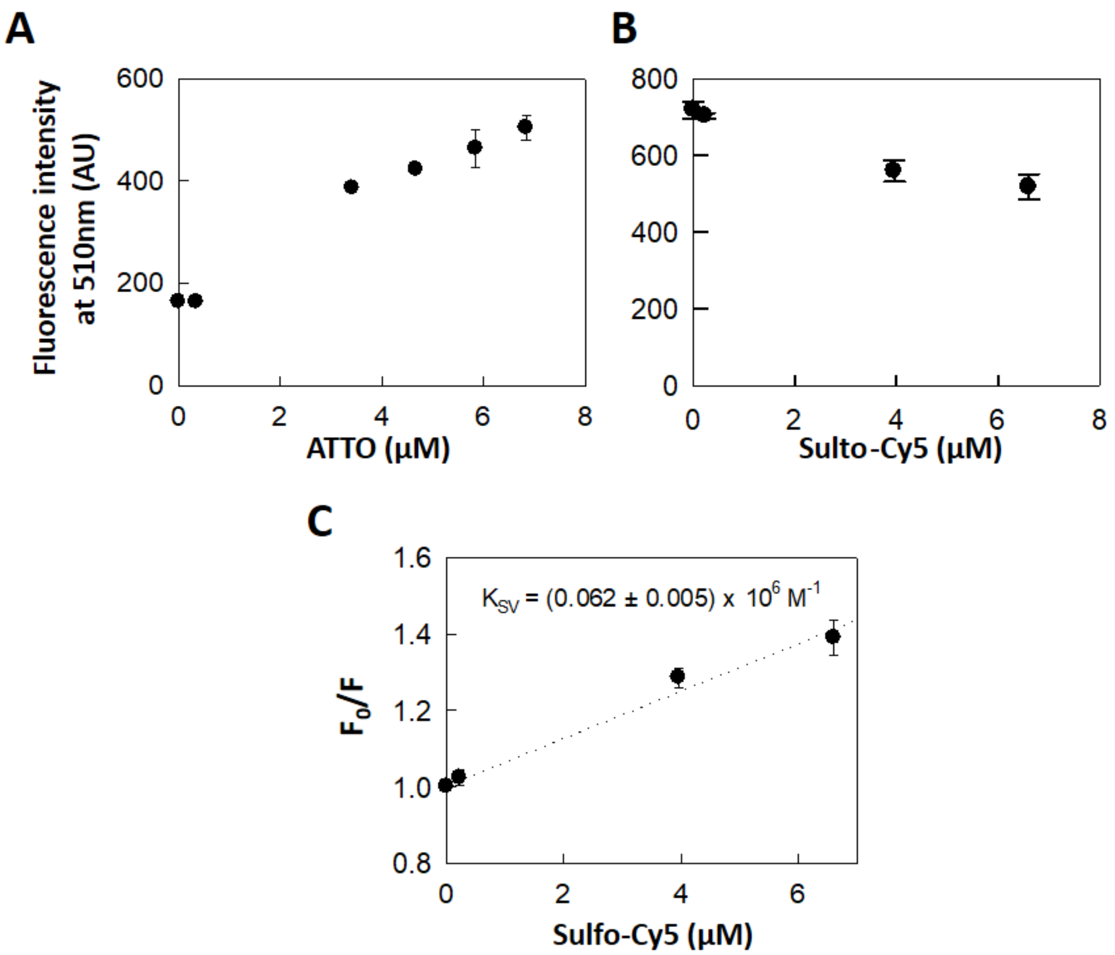

Figure S3. Fluorescence dye mediated GFP quenching analysis. **A**) Fluorescence emission intensity of T22-GFP-H6 in presence of increasing concentrations of free ATTO molecules. **B**) Fluorescence emission intensity of T22-GFP-H6 in presence of increasing concentrations of free Sulfo-Cy5 molecules. **C**) Stern Volmer Plot of T22-GFP-H6 quenching in presence of increasing concentrations of free sulfo-Cy5 molecules. Stern-Volmer constant (K_{SV}) is indicated in the plot and estimated from a linear regression analysis.

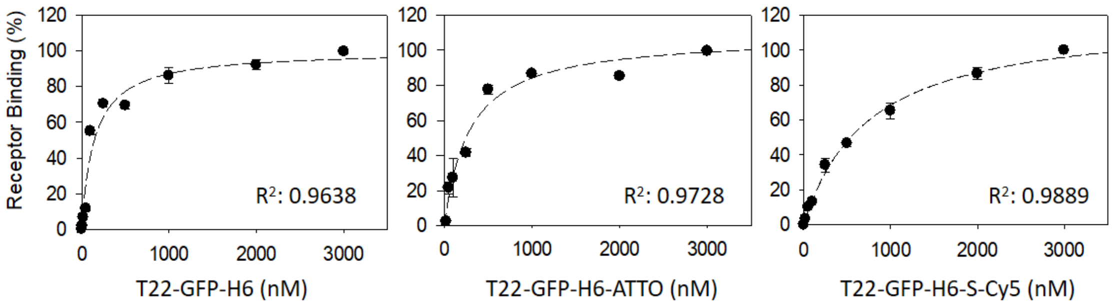

Figure S4. Nanoparticles specific binding to the CXCR4 receptor. T22-GFP-H6, T22-GFP-H6-ATTO and T22-GFP-H6-S-Cy5 binding to CXCR4+ Toledo cells after 1h of incubation at 4 °C. Graph represent CXCR4-specific binding calculated by Total protein binding–unspecific binding at each nanoparticle concentration. Data is shown as receptor binding average ± standard error.

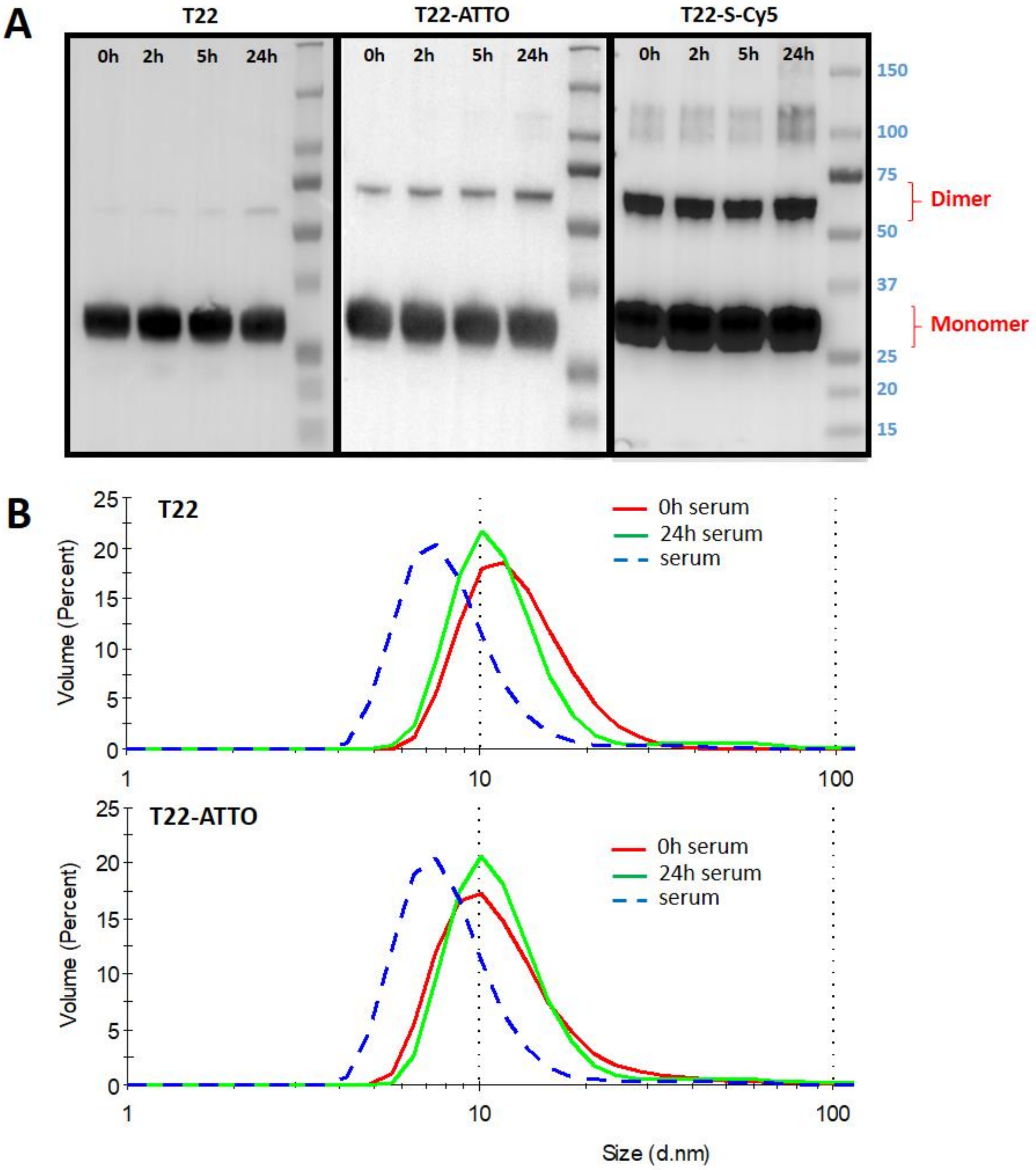

Figure S5. Nanoparticles stability in human serum: **A**) Proteolytic stability of T22-GFP-H6 (T22), T22-GFP-H6-ATTO (T22-ATTO) and T22-GFP-H6-S-Cy5 (T22-S-Cy5) nanoparticles in human serum at different incubations times up to 24 h analyzed by wester-blot immunodetection with a monoclonal anti-His antibody (Santa Cruz Biotechnology). Molecular weight marker is indicated in KDa. **B**) Volume size distribution of nanostructured T22-GFP-H6 (T22) and T22-GFP-H6-ATTO (T22-ATTO) at 0 h and 24 h after incubation in human serum. Measurement of control serum is depicted in dashed lines. T22-GFP-H6-S-Cy5 nanoparticles cannot be analyzed by DLS since Sulfo-Cyanine 5 fluorescence prevent reliable light scattering measurements.

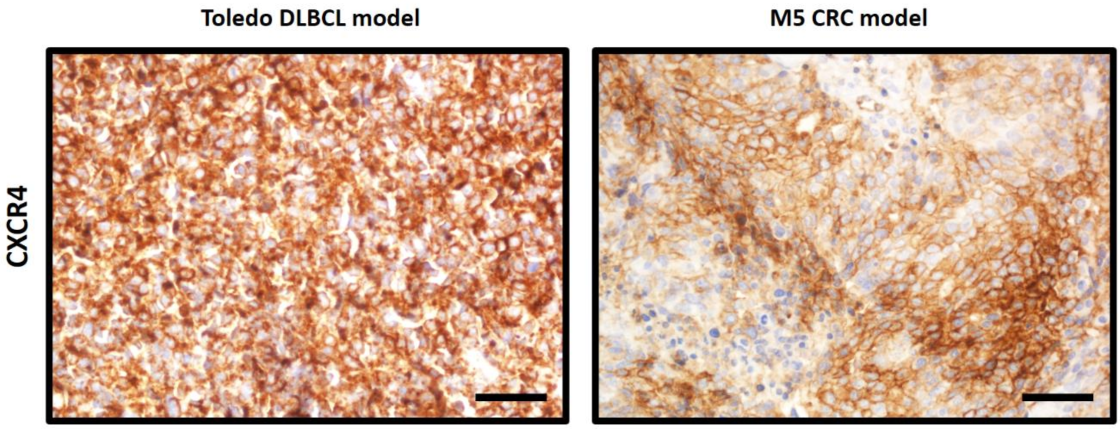

Figure S6. CXCR4 expression in DLBCL and CRC models: the level of CXCR4 receptor overexpression, as detected by Immunohistochemistry (IHC), is significantly higher in subcutaneous tumors derived from Toledo DLBCL cell line than in SC tumors derived from the M5 CRC tumor line. Scale bar indicates 50 µm.

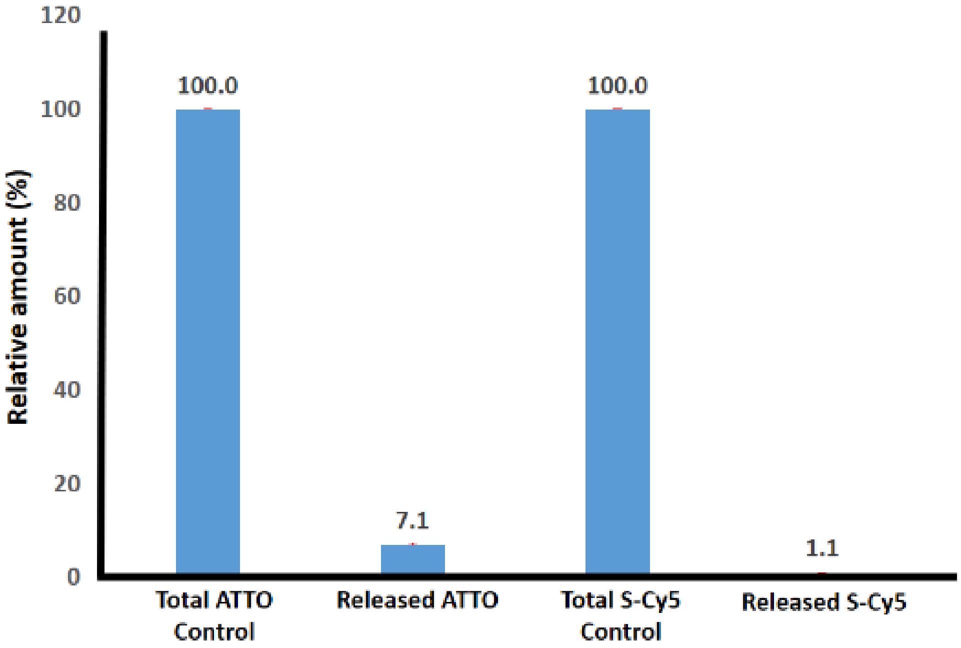

Figure S7. Fluorescent dye leakage in human serum: Fluorescence signal percentage in dialysis buffer upon dialysis of Free ATTO488 and S-Cy5 molecules or human serum incubated T22-GFP-H6-ATTO and T22-GFP-H6-S-Cy5 nanoconjugates. Each nanoconjugate fluorescence signal is relative to its respective free fluorochrome fluorescence.

Table S1. Biodistribution in subcutaneous DLBCL mouse model 5 h post administration represented as percentages of total fluorescence uptake (% TFU) in different organs. Significant differences in organ uptake between T22 and T22-ATTO or T22-S-Cy5 are indicated as * at $p < 0.05$

DLBCL 5h (% TFU)	Tumor	Kidney	Liver	Lung	Pancreas	Spleen
**T22**	74 ± 5	0.2 ± 0.2	2 ± 2	3 ± 3	20 ± 6	0.8 ± 0.8
**T22-ATTO**	19 ± 2 *	37 ± 3 *	29.5 ± 0.9 *	2.5 ± 0.2	11 ± 1	0.7 ± 0.4
**T22-S-Cy5**	17 ± 2 *	36 ± 4 *	26.6 ± 0.9 *	14 ± 3 *	5.6 ± 0.4 *	1.3 ± 0.7

Table S2. Biodistribution in subcutaneous CRC mouse model 5 h post administration represented as percentages of total fluorescence uptake (% TFU) in different organs. Significant differences in organ uptake between T22 and T22-ATTO or T22-S-Cy5 are indicated as * at $p < 0.05$.

CRC 5h (% TFU)	Tumor	Kidney	Liver	Lung	Pancreas	Spleen
**T22**	57 ± 7	8.2 ± 0.9	6.4 ± 0.4	6.3 ± 0.1	20 ± 3	1.9 ± 0.3
**T22-ATTO**	22.9 ± 0.8 *	30 ± 1 *	32.5 ± 0.9 *	4 ± 3	9 ± 4	0.5 ± 0.2 *
**T22-S-Cy5**	20 ± 8 *	11.0 ± 0.5	54 ± 12	5.1 ± 0.6	3.5 ± 0.2*	7 ± 2

References

[1] Hughes L.D., Rawle R.J., Boxer S.G. Choose your label wisely: water-soluble fluorophores often interact with lipid bilayers. PloS one 2014; 9:e87649.
